# Ab initio detection of fuzzy amino acid tandem repeats in protein sequences

**DOI:** 10.1186/1471-2105-13-S3-S8

**Published:** 2012-03-21

**Authors:** Marco Pellegrini, Maria Elena Renda, Alessio Vecchio

**Affiliations:** 1 Istituto di Informatica e Telematica, CNR - Consiglio Nazionale delle Ricerche, Pisa I-56124, Italy; 2 Dipartimento di Ingegneria dell'Informazione, Università di Pisa, Pisa I-56122, Italy

## Abstract

**Background:**

Tandem repetitions within protein amino acid sequences often correspond to regular secondary structures and form multi-repeat 3D assemblies of varied size and function. Developing internal repetitions is one of the evolutionary mechanisms that proteins employ to adapt their structure and function under evolutionary pressure. While there is keen interest in understanding such phenomena, detection of repeating structures based only on sequence analysis is considered an arduous task, since structure and function is often preserved even under considerable sequence divergence (*fuzzy tandem repeats*).

**Results:**

In this paper we present *PTRStalker*, a new algorithm for *ab-initio *detection of *fuzzy tandem repeats *in protein amino acid sequences. In the reported results we show that by feeding *PTRStalker *with amino acid sequences from the UniProtKB/Swiss-Prot database we detect novel tandemly repeated structures not captured by other state-of-the-art tools. Experiments with membrane proteins indicate that *PTRStalker *can detect global symmetries in the primary structure which are then reflected in the tertiary structure.

**Conclusions:**

*PTRStalker *is able to detect fuzzy tandem repeating structures in protein sequences, with performance beyond the current state-of-the art. Such a tool may be a valuable support to investigating protein structural properties when tertiary X-ray data is not available.

## Introduction

### Motivations

In a seminal paper of 2001 M.A. Andrade and co-authors [1] observe that repetitive subsequences that appear tandemly often form integrated assemblies when viewed in their three-dimensional corresponding conformation, as they often confer multiple binding opportunities and play a structural role in proteins. Moreover, tandemly repeated structures constitute a different class from *domains *and *motifs *that appear singly in each protein (while they can be repeated across families of protein). M.A. Andrade and co-authors also remark that repeats in protein sequences are usually hard to detect because of their relatively (on average) short length and because of their considerable -inherent- sequence divergence.

A study of 1999 by Marcotte et al. [2] indicate that internal subsequence repetitions in proteins primary structure are quite widespread. They have been noticed in about 14% of all the known proteins, with eukaryotic proteins being three times more as likely to have internal repeats than prokaryotic ones. The distribution of TRs in protein families and the mechanisms of protein TR formations are discussed in detail in [3].

From a theoretical point of view almost any approach that works for a 4-characters alphabet (DNA) could also be applied to the 20-characters amino acid alphabet and indeed some methods are developed for "generic" biological sequences (e.g. [4]). In practice, there are differences between protein and DNA sequence, and the way they can be analyzed. Protein sequences tend to be shorter (at most of the order of 10^4 ^amino acids) than DNA sequences (often of the order of 10^6 ^nucleotides and more). In proteins, assigning a score to an amino acid substitution is important, and finally repetitions in amino acid sequences tend to be very divergent, since the same structural role can be maintained even with little sequence conservation. Also, empirically, we observe that the tools for detecting (tandem) repeats for DNA and for proteins constitute two rather distinct families, often employing completely different characterization and algorithmic approaches.

In [5] we presented TRStalker, an algorithm designed, developed and tuned to detect fuzzy tandem repeats in DNA sequences. In view of the biological relevance of tandemly repeated subsequences in proteins, we worked on an evolution of TRStalker, modified and tuned to work with protein sequences in order to detect amino acid (aa) tandem repeats, by incorporating amino acid similarity information deep into the algorithmic framework. Amino acid substitution matrices (such as the PAM and BLOSUM families of matrices, based on known evolutionary changes of amino acids in proteins) are usually expressed in terms of a *similarity score *between amino acids. In the framework we present here, we use the notion of weighted edit *distance *between sequences, thus converting standard similarity scores into distance weights. Interest in metric-space search and indexing in bioinformatics [6] has been recently rekindled since one can tap on a vast array of efficient metric-based generic methods developed over the years, thus reducing the need for developing ad-hoc searching and indexing algorithms for each new similarity measure. A technique for turning BLOSUM log-likelihood similarity matrices into corresponding metric space was proposed in [7], while a second method to turn PAM log-likelihood similarity matrices into a corresponding metric space was proposed in [8]. It is also possible to define new metrics over amino acids by first mapping each amino acid into a real vector space (see e.g. [9]), and then applying one of the many possible metrics associated to a real vector space. For this reason we generalize the definition of tandem repeat in order to incorporate this larger class of metrics in the proposed algorithm.

*PTRStalker*, the novel algorithm we developed, has been compared against competing state-of-the-art methods over large collections of proteins (UniprotKB/Swiss-Prot), on membrane channels proteins, and on selected families of proteins known to contain long fuzzy TRs. We found out that under edit distance 19.53% of the protein listed in UniPtotKB/Swiss-Prot have significant fuzzy TRs. We could find fuzzy TRs, not detected by some competing methods (XSTREAM, T-REKS, TRUST), in Human Titin (striated muscles). For the Chlorine channel protein ClC-0 (membrane transport) we show that PTRStalker can detect known symmetries while competing methods do not. The output of our analysis of protein sequences has been collected into a publicly available database, that will be continuously updated in order to provide useful insight to researchers interested in protein sequence analysis. The database is freely available at [10].

The outline of the paper is the following: after a short introduction on the algorithms developed for detecting tandem repeats in protein sequences, we introduce and describe PTRStalker procedural approach in detail. Next we describe the experiments we performed to evaluate the metric chosen, and the results obtained by PTRStalker and competing state of the art tools.

### State of the art

The first methods developed for finding TRs in proteins are based on detecting sub-optimal alignments in the self-alignment matrix generated by the Smith-Waterman algorithm. Some examples are Internal Repeat Finder [2,11], RADAR [12], REPRO [13,14] and TRUST - Tracking Repeats Using Significance and Transitivity [15], even if they often detect both tandem and interspersed repeats. More recently Newman and Cooper proposed XSTREAM [16], which uses a seed expansion approach, while Jorda and Kajava proposed T-REKS [17], which uses a clustering approach based on k-means. The systems HHrep [18] and HHRepID [19] are instead based on building and matching Hidden Markov Models for the repeating substrings to be sought (not necessarily tandem). Some approaches based on neuronal networks aim at detecting particular repetitive structures. For example, in [20], Palidwor et al. developed a classification technique for detecting alpha-rods repeats, a specific repetitive structure. A meta searching approach that combines the output of different algorithms has been also proposed, for example in the tool REPPER [21]. With a few notable exceptions (see e.g. [4]) all these methods specialize on protein sequences. Sokol et al. [22] proposed TRED, which is based on an estimation of edit distance between strings. In principle their approach can be applied to any alphabet size, however the on line available software is tuned only for 4-character DNA strings.

Among the -few- databases reporting protein repetitive subsequences we recall ProtRepeatsDB [23], a relational database of perfect and mismatch repeats, which also provides cross species comparisons of different types of amino acid repeats.

## Methods

### Algorithm PTRStalker

In this Section we describe PTRStalker, an evolution of the algorithm TRStalker [5] we developed for finding TRs within DNA sequences. Whereas TRStalker and PTRStalker use the same overall procedural approach to detect tandemly repeated subsequences, PTRStalker has been opportunely tuned for detecting TRs in protein sequences. In particular, the main differences are in the formal definition of a tandem repeat, in the metrics used, and in the q-grams matching policy (see below). However, the overall idea still holds: TRs are detected by *(i) *first finding a set of candidate periods, *(ii) *then finding a set of candidate pairs (period, starting position), and *(iii) *finally verifying if in a particular position there exists a TR according to the precise mathematical definition adopted (see below). To make this paper self contained, after introducing a few working definitions we will recall the general structure of the algorithm presented in [5], describing the innovative parts PTRStalker has.

### Preliminaries

In order to extend the methodology used in [5] by incorporating amino acid similarity information encoded in similarity matrices, we need to perform two tasks: define a *weighted edit distance*, and suitably modify the tandem repeat definition so to obtain a *Weighted Steiner Simple Tandem Repeat *(Weighted Steiner STR). We give here details for the BLOSUM metric space as defined in [7]. The extension to other metrics is straightforward.

#### BLOSUM-weighted edit distance

We incorporate the definition of BLOSUM-derived edit distance between two strings as follows: the cost of indel operations is equal to 1, while the cost of substitutions depends on the involved characters. Since BLOSUM matrix entry *M_ij _*defines the level of similarity between characters *i *and *j*, and not their distance, the cost *C_ij _*of a substitution between characters *i *and *j *is computed, according to [7], as the ratio:

$$C_{ij}=\frac{D_{ij}}{D_{max}}$$

where

$$D_{ij}=M_{ii}+M_{jj}-2M_{ij},$$

$$D_{max}=\underset{{{}_{i}}_{\in \Sigma ,j\in \Sigma }}{\text{max}}D_{ij}$$

and ∑ is the amino acid alphabet. Since *D_ij _*represents the non-normalized distance between two characters, we use *D_max_*, representing the maximum distance between two characters, as the normalization value so to obtain the normalized weight *C_ij _*∈ [0, 1]. In [7] it is shown that these weights are consistent, since they almost always satisfy the triangular inequality.

#### BLOSUM-weighted Steiner-STR

Let *D_B_*(*a*, *b*) be the BLOSUM-derived edit distance between strings *a *and *b *computed by using the BLOSUM-derived weights defined above. In order to give meaningful limits to the divergence of strings under this metric we need to define its behavior for random pair of amino acid strings. For the expected edit distance of two strings no analytic formula is known even in the unweighted case. Even if the theory of Karlin and Altshul [24] could give some insight, the issue of extending their methodology to the case of tandem sequences is not trivial. For these reasons we estimate *D_B_*(*a*, *b*) with an experimental approach, by generating a suitable number of random strings in which the probability of amino acid substitution is consistent with the BLOUSM data, and by measuring the weighted edit distance for a given error level. We define with *E*(*C*) the expected substitution cost among two amino acids due to the cost matrix *C*. As a consequence, two random amino acid stings of length *p *= |*a*| = |*b*| are at expected distance *pE*(*C*). If we allow only a percentage *μ *of substitutions we obtain an estimated weighted distance of *μpE*(*C*).

A *BLOSUM-weighted Steiner-STR *is defined as a string *X *= *x*_1_*x*_2_..*x_t _*for which two conditions hold, for a user defined error parameter 0 ≤ *μ *≤ 1, and constant *c *with 1 ≤ *c *≤ 2:

(a) for each *i *∈ [1, .., *t *- 1], *D_B_*(*x_i_*, *x_i_*_+1_) ≤ *cμ*|*x_i_*|*E*(*C*)

(b) there exists a Steiner string $\bar{x}\in \Sigma^{*}$ so that for each *i *∈ [1, .., *t*], $D_{B}\left(\bar{x},x_{i}\right)\leq \mu |x_{i}|E\left(C\right)$

Intuitively, in a BLOSUM-weighted Steiner-STR the TR consists of *t *duplications of a single Steiner consensus string $\bar{x}$ with at most *μ *times the number of mutations one would expect from random strings of the same length (condition (b)). Moreover consecutive copies of the mutated string do not diverge too much w.r.t. each other, at most *cμ *times the number of mutations one would expect from random strings of the same length (condition (a)). Note that condition (a) is vacuous for *μ *≥ 1/*c*. The choice for the constant *c *depends also on the level of divergence. For protein repeats with low divergence *c *= 2 is a sensible choice since two copies at distance *μ*|*x_i_*|*E*(*C*) from $\bar{x}$ are also at distance at most $2\mu |\bar{x}|E\left(C\right)$ from each other by the triangular inequality. Thus (a) is a necessary condition for (b). For the higher level of divergence we are interested in (*μ *= 0.3), the value *c *= 2 is too loose and we use a lower value (*c *= 1.5), so to maintain a good filtering ability of condition (a) and to avoid having as a possible solution a TR where the consecutive pairs may have a very irregular divergence. Note that the standard TR definition for the unweighted edit distance corresponds to a matrix with cost 1 for each substitution.

#### Homologous q-grams

Let *I *be a finite subset of non-negative integers. We call *I *an *index set*. The *span *of *I *is *span*(*I*) = *max*{*i *- *j*|*i*, *j *∈ *I*}, the position of *I *is *pos*(*I*) = min *i *∈ *I*, and the *shape *of *I *is *shape*(*I*) = {*i *- *pos*(*I*)|*i *∈ *I*}. When |*I*| = *q *and *span*(*I*) = *s*, the shape of *I *belongs to the class of (*q*, *s*)-shapes. Any set of non-negative integers *Q *containing 0 is a shape. For an alphabet ∑ (the 20 amino acid letters in our case), a string *S *∈ ∑* of length *n *can be seen as a function defined over [0, ..,*n *- 1] with values in ∑, and for any subset *I *⊂ [0, .., *n *- 1] the restriction of *S *to *I*, denoted by *S*[*I*], is a substring of *S*. Given any shape *Q *in the class of (*q*, *s*)-shapes, all sets *I *⊂ [0, ..*n *- 1] such that *shape*(*I*) = *Q*, form the set of *Indexes*(*Q*, *n*). We can use elements from the *Indexes*(*Q*, *n*) to generate restrictions for the string *S*. An index set *I *such that |*I*| = *q *and *span*(*I*) = *q *- 1 is called an *ungapped q-gram *since its shape is *shape*(*I*) = [0, ..*q *- 1]. If we have an index set *J *with |*J*| = *q *and *span*(*J*) = *s *≥ *q *we have a *gapped q-gram *since its shape is formed of non consecutive integers. As noted in [25] gapped q-grams are strictly more powerful than ungapped ones. In order to generate a population of candidate periods we consider now all possible (*q*, *s*)-shapes with *q *= 3 and *s *= 4, 3, 2. Denoting with - the gaps and with # symbols from ∑, (the first and last positions must be always #), we have the (3, 4)-shapes ## - - #, # - # - #, and # - - ##; the (3, 3)-shapes # - ##, ## - #; the (3, 2)-shape ###; and the (2, 1)-shape ##.

Gapped q-grams (also called *spaced seeds*) have been used in [26-28] to speed up homology search. In these papers much larger values of *q *and *s *are used in order to attain sensitivity. Therefore the key problem for them becomes how to select one (or a few) effective seed out of an exponentially large family (for *s *and *q*). Since we use small values of (*q*, *s*) we do not have this seed selection problem and we can afford to use a complete family, formed by 6 seeds only.

In defining the notion of *homologous q-grams *for amino acid sequences we take into account the fact that functional properties of proteins are preserved even under considerable sequence substitutions. The BLOSUM similarity matrices give a quantitative definition of the allowed amino acid substitutions. Given two index sets *I*_1_, *I*_2 _∈ *Indexes*(*Q*, *n*), we call them *matching *(or *homologous *in *S*, if *S*[*I*_1_] and *S*[*I*_2_] have two identical symbols in corresponding positions while the symbol in the remaining position can be different in the two strings but within ranking *z *of each other as given by the BLOSUM similarity matrix (that is, the one symbol is among the top *z *most similar symbols to the other one, and vice versa). The slackness parameter is *z *= 1 when we want exact match, and *z *= 3 and *z *= 5 when we allow more substitutions. The value |*pos*(*I*_1_) - *pos*(*I*_2_)| is called the *period *of the match.

Note that, by considering the *z *amino acids closer to a given amino acid, we introduce a discrete ranking in the metric space. Alternatively one could choose a fixed radius and consider all amino acids within that radius. We performed dedicated experiments in order to choose the right approach and the results (not shown here) indicate that there is almost no difference in the two approaches. We decided to adopt the discrete ranking approach because choosing suitable radii implies ad-hoc dependance on the specific metric.

#### Anti-smear weighting

The anti-smear weighting technique allows to cope with the fluctuations in the period of matching q-grams introduced by insertion and deletions of amino acids in a sequence. If *q*_1 _and *q*_2 _are occurrences of homologous *q*-grams in *X *at distance *k*, before the implant of mutations, the effect of insertion and deletions on the positions of the string *X *between *q*_1 _and *q*_2 _is to alter their distance so that a different period *k*' is detected. The difference *k *- *k' *is equal to the algebraic sum of number of insertions and deletions in the positions between *q*_1 _and *q*_2_. Assuming that any such position can be an insertion or a deletion independently with the same probability, the random variable *k *- *k*' is distributed as a sum of independent r.v. with values in {+1, -1, 0} with mean value 0, thus, by a Chernoff bound argument, its tail distribution decays exponentially [29,30]. Also near-by probes in *X *have small variations in the value of the shift *k *- *k*'. Inspired by the above observation we devise a weighting scheme that increments the total weight of period *k *if another period of value $\bar{k}$ is discovered in a near-by position, with weights that decay exponentially with $\left|k-\bar{k}\right|$.

Let *q *be a *q*-gram in the input string *Y *at position *i*. Let *q*_1_, .., *q_h _*be the next *h *occurrences of *q *in *Y *following the occurrence at position *i*. The *h *corresponding detected distances are *k_g _*= *j_g _*- *i*, for *g *∈ [1, ..*h*]. For the period *k_g_*, we increment its weight:

(1) $$w_{0}\left(k_{g}\right)=w_{0}\left(k_{g}\right)+\underset{k\in Q}{\sum }2^{-|k_{g}-k|},$$

where *Q *is a queue holding the last *H *detected distances in the sequential scan of the input string *Y*. This way, the final weight *w*_0_(*k*) for a given period *k *is the sum of the individual anti-smear weights computed above for probes at distance *k*. After the weight update, we enqueue all *h *values *k_g _*in the queue *Q*, and we dequeue an equal number *h *of items.

#### Multiplicity weighting

The goal of this technique is to strengthen the signal when the TR is made by more than two repeating units. Let *w*_0_(*k*) be the weight of the period *k *as assigned by the anti-smear weighting procedure. As observed before for a TR with a large number of copies we will find also integer multiples of *k *with a relatively high frequency. We take advantage of this fact and compute new weights:

(2) $$w_{1}\left(k\right)=\underset{h\geq 1}{\sum }w_{0}\left(hk\right).$$

The candidate periods are then sorted by the weight *w*_1_(.), and processed in decreasing order.

#### Positional k-density

We further exploit the property of TRs for which the same period is detected by probes in near-by positions. The *positional k-density *is the density of probes that contribute to the counter for the candidate period *k*. Let *k *be the period under investigation. Consider the set *K_k _*of the positions of those *q*-grams (i.e. substrings of *Y*) that contribute to the weighting of *k *through the multiplicity weighting. In order to avoid double counting we always take the position of the first of the two matching probes. Note that, if a position is shared by several pairs of probes it will be counted only once. Let *f *: [0, ..,|*Y*| - 1] → {0, 1} the characteristic function that for each position in *Y *denote the membership of that position to *K_k_*. Consider the *k*-window smoothing of $f:F\left(i\right)=\sum_{j=i}^{i+k-1}f\left(j\right)$ that computes the *k*-smoothed density of the function *f*, for *i *∈ [0, .., |*Y*| - *k*]. Finally we define a threshold *t*(*k*) proportional to the average *k*-density by a user-defined constant, and we consider as a candidate position set *CP*(*Y*, *k*) = {*i *∈ [0, .., |*Y*| - *k*]|*F*(*i*) ≥ *t*(*k*)}. The output of this positional density computation is a sequence of pairs (*k*, *i*) where *k *is a candidate period and *i *a candidate position.

#### TR validation

We take each candidate pair (*p*, *i*) and explicitly test whether there is a TR of period *p *starting in position *i *according to the definition used, by using an alignment technique based on the well-known Smith-Waterman algorithm. In this phase, besides validating the TRs, we discover the (fractional) repetition number of the TRs eventually extracted. Finally, we check for inclusion the TRs found and we filter out those TRs completely enclosed in another one. For TRs in the same position and length but different period we report the TR with shorter period.

### The algorithm in detail

**Figure 1:**
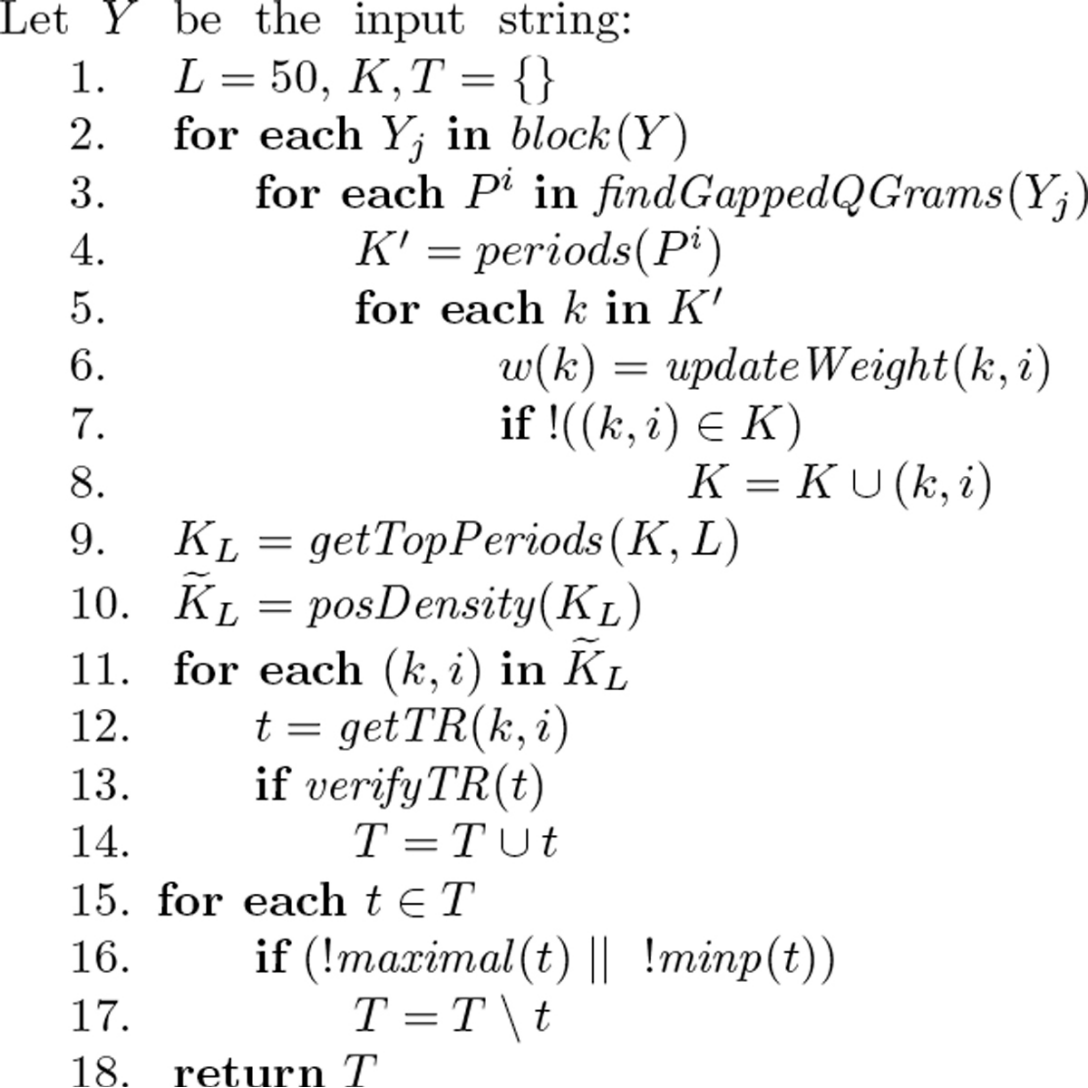
PTRStalker algorithm scheme.

In the following we will go through the high level pseudocode of PTRStalker, reported in Figure 1, explaining each phase and function in detail.

**Step 1**. The number *L *of candidate periods to examine is empirically set to 50, while the set of candidate pairs *K*, and the set of TRs *T *to be given as output are initialized as empty sets.

**Step 2**. The function *block*(*Y*) splits the input sequence *Y *into *n *blocks *Y_j_*, 1 ≤ *j *≤ *n*, of predefined length (we used a value of 2000 aa). We limit our computations within a block so to avoid counting q-grams when they are too far to be involved in a TR local to the block. For TRs stranding across blocks we adopt mechanisms to carry over useful information from one block to the next one.

**Steps 3-4**. The candidate periods are found by detecting the distance (counted in amino acid positions) among *homologous q-grams*. Because of the presence of substitutions, insertions, and/or deletions, many instances of q-grams will be probably affected by error and a match could be missed, thus reducing the frequency counts for the candidate period *k*. In order to cope with this effect, for each block *Y_j_*, function *findGappedQGrams*(*Y_j_*) records for each occurrence of a *gapped q-gram P^i^*in *Y *its distances *K*' to the next 5 occurrences (candidate periods) and its starting position *i *in *Y*. Note that the candidate periods will be processed later in order of cumulative weight.

**Steps 5-8**. For each period *k *detected by a q-gram at position *i*, the function *updateWeight*(*k*, *i*) increments the weight *w*(*k*) of the period *k *∈ *K*' by applying the two weighting techniques presented before: the anti-smear weighting technique to cope with the fluctuations in the period of matching q-grams introduced by insertion and deletions of amino acids in a sequence (see Equation 1), and the multiplicity weighting (see Equation 2) to strengthen the signal when the TR is made by more than two repeating units. For computing the anti-smear weight as shown in Equation 1 we empirically set *h *= 5 and *H *= 20. The candidate pair (*k*, *i*) is then added to the set *K*, if it is not already present.

**Step 9**. Function *getTopPeriods*(*K*, *L*) ranks the periods by *weighted frequency *and returns only the top *L *positions in the set *K_L_*.

**Step 10**. PTRStalker further exploits the property of TRs that the same period is detected by probes in near-by positions through the positional *k*-density. Thus, the function *posDensity*(*K_L_*) computes the density of probes that contribute to the counter for the candidate period *K*, and then cuts off for position with low density, returning those candidates with higher positional density.

**Steps 11-14**. For each candidate pair (*k*, *i*), functions *getTR*() computes a candidate TR of period *k *starting at position *i *and *verifyTR*() verifies whether there is a tandem repeat *t *of period *k *starting at position *i *according to the definition of TR given, and if so *t *is added to the set *T*. Recall that for a BLOSUM-weighted Steiner-STR we set *μ *= 0.3 and *c *= 1.5.

**Steps 16-18**. Finally, for each candidate tandem repeat *t *∈ *T *the function *maximal*() verifies whether *t *is included in a longer TR, and possibly removes *t *from *T*, while *minp*() for TRs in the same position and length but different period maintains only the TR with shorter period. The procedure returns the set *T *as result.

The elements of *T *can be visualized and listed according to different properties of the TR found: initial position, final position, repeating unit size, number of repetitions, total length, absolute divergence, mean divergence, etc.

## Results

In this section we report PTRStalker performance with the use of a BLOSUM-based metric and the unweighted edit distance. Furthermore we report a detailed comparison of PTRStalker versus some state of the art algorithms. For some of the experiments reported below, PTRStalker has been run to analyze the UniprotKB/Swiss-Prot database of protein sequences. More in detail, the version we used in our experiments was UniProtKB/Swiss-Prot Release 57.15 of 02 March 2010 -henceforth called Swiss-Prot database, that contains 515, 203 sequence entries.

### Metric evaluation

We performed a set of experiments to evaluate the effects of BLOSUM-based metrics and q-gram similarity. For this reason PTRStalker has been run on the first one thousand sequences of the database, using the definition of BLOSUM-weighted Steiner-STR. We also evaluated the influence of the q-gram similarity matching parameter *z*. Table 1 reports the percentage of sequences that contain at least a TR with length ≥ 20 when using edit distance and BLOSUM-based distance (three different matrices have been used: BLOSUM 90, BLOSUM 70, and BLOSUM 50). The table also show the results obtained for different levels of q-gram similarity. When *z *= 1 similarity is not considered: a q-gram is only similar to itself. When *z *= 3, for every q-gram also the two other more similar q-grams have been considered (the total number is three), and so on. Tables 2, 3 show the results obtained when considering TRs with length ≥ 30, and ≥ 40. The maximum sensitivity is attained for almost all length classes with *z *= 5 and the BLOSUM 50 matrix. The use of BLOSUM metrics more than doubles the sensitivity for TR of length above 20 aa w.r.t the unweighted edit distance. Experiments shown later indicate that for the chosen parameters, the threshold 20 is far from the expected maximum TR length in shuffled sequences (about 15) for sequences of length at most 1000, which constitute 99,96% of the sequences in UniProtKB/Swiss-Prot. These experiments indicate that the use of BLOSUM based metrics within PTRStalker is effective in detecting long fuzzy TRs.

**Table 1:** UniProtKB/Swiss-Prot database: percentage of protein sequences that contain at least a tandem repeat with length ≥ 20

*Metric*	*z = 1*	*z = 3*	*z = 5*
BLOSUM 90	17.7	20.5	23.2
BLOSUM 70	21.6	25.6	26.1
BLOSUM 50	22.5	27.4	29.1
Edit	4.3	-	-

**Table 2:** UniProtKB/Swiss-Prot database: percentage of protein sequences that contain at least a tandem repeat with length ≥ 30

*Metric*	*z = 1*	*z = 3*	*z = 5*
BLOSUM 90	5.7	6.4	7.8
BLOSUM 70	7.0	7.0	8.7
BLOSUM 50	7.1	8.4	8.8
Edit	2.2	-	-

**Table 3:** UniProtKB/Swiss-Prot database: percentage of protein sequences that contain at least a tandem repeat with length ≥ 40

*Metric*	*z = 1*	*z = 3*	*z = 5*
BLOSUM 90	4.3	4.9	4.9
BLOSUM 70	4.9	5.5	5.4
BLOSUM 50	4.9	5.7	5.7
Edit	1.8	-	-

### Competitors

In what follows we compare PTStalker with several state of the art algorithms, namely, RADAR, TRUST, XSTREAM, T-REKS, HHrep and HHRepID. Like PTRStalker, all these algorithms do ab-initio identification of protein repeats with indels.

RADAR [12] and TRUST [15] detect internal sequence symmetries by aligning the protein sequence to itself and analyzing the collection of suboptimal alignments. In particular, they build a repeat profile to exactly determine repeat borders and extract a multiple alignment of repeats. Usually, the alignment of a sequence to itself for the detection of suboptimal alignments is performed with the Smith-Waterman algorithm [31] or its variations (see, e.g., [32]).

HHrep [18] and HHRepID [19] are based on HMM-HMM comparison instead of sequence-sequence comparison: a Hidden Markov Model (HMM) profile is built from a multiple alignment of proteins homologous to the analyzed one, and sub-optimal alignments are then searched by aligning this HMM with itself. These methods can be very sensitive in detecting long and highly divergent repeats.

TRUST and HHrep make also use of the concept of transitivity of alignments, through which identify additional distant homologue TRs and recognize and reduce the contribution of non-homologous TRs.

XSTREAM [16] is based on a short string extension algorithm.

T-REKS [17] uses a K-means clustering algorithm for identifying putative lengths of TRs. XSTREAM and T-REKS use quite similar definition of TR and are especially effective in finding relatively short tandem repeats (15-20 bp long).

### Proteins with very long tandem repeats

In this experiments we compared PTRStalker with two state of the art algorithms: XSTREAM [16] and TREKS [17], for the task of detecting very long tandem repeats (spanning more than 4000 aa). We also tested two algorithms TRUST [15] and RADAR [12] which produce clusters of generic (interspersed) repeated motifs. We have filtered the output of TRUST and RADAR in order to highlight the TRs found. Entries in the RADAR column of table 4 are marked "***" when there is no evident TR cluster in the output, although many interspersed repeats may be found. HHRep and HHRepID are less suitable for this task since they report pairs of homologous substrings, thus failing to report multi-repeating units. We have selected 12 proteins from Swiss-Prot database for which a tandem repeat of length ≥ 4000 aa has been detected by PTRStalker. The data in Table 4 show that T-REKS with the standard parameter setting for these long proteins does not detect fuzzy TRs longer than 100 aa in 6/12 cases (marked "*"), and, even when longer TRs are found, these are often sub-TR of those found by the other methods. PTRStalker and XSTREAM have a remarkable consistence in detecting the location of the longest fuzzy TR in 11/12 cases. Sometimes they differ in the periodicity, since XSTREAM gives priority to higher repeat number (and consequently shorter period), while PTRStalker prefers TR with longer span (often attained with a lower copy number and longer period). One notable difference in the output of PTRStalker and XSTREAM is for the Human Titin sequence (involved in the functioning of vertebrate striated muscles). This protein is one of the longest and most complex human proteins. Here PTRStalker is able to detect a much longer TR (4-repeat, 1082-period) completely missed by the other two methods, RADAR also found a long TR structure in the same region. The domain composition of Human Titin has been analyzed in [33,34]. The long fuzzy TR we have found falls in the A-band region, which is known to contain two long super-repeating patterns both composed of regular patterns of Ig and FN-III motifs [34]. In order to appreciate the unusual length of this Fuzzy TR we also performed an experiment by feeding PTRStalker with a random shuffled Titin sequence, in which the longest fuzzy TR found (with the same configuration parameters) has length 25. For 6 sequences out of 12, RADAR did return large clusters of interspersed repeats covering the region containing the TR, while for 6 sequences the cluster produced corresponds to a long TR. In general, we can conclude that PTRStalker is at a par with XSTREAM and RADAR for detecting this class of long fuzzy TRs, and better than T-REKS (in output quality) and TRUST (in efficiency).

**Table 4:** Analysis of the 12 proteins from the UniProtKB/Swiss-Prot database with a very long fuzzy tandem repeat

Protein			Tandem repeats found by				
**Acc #**	**ID**	**Length**	**PTRStalker**	**XSTREAM**	**T-REKS**	**TRUST**	**RADAR**

Q8IVF2	AHNK2_HUMAN	5795aa	165-x-24	165-x-23	*	**	163-x-31
			[720-4666]	[774-4617]			[289-5529]
Q9N4M4	ANC1_CAEEL	8545aa	915-x-6	903-x-4.27	58-x-4	**	***
			[3000-8491]	[4342-8199]	[2336-2567]		
P08519	APOA_HUMAN	4548aa	1495-x-3	114-x-37	114-x-24	114-x-39	111-x-38
			[0-4486]	[7-4220]	[1501-4125]	[18-4523]	[17-4282]
P20930	FILA_HUMAN	4061aa	1339-x-3	323-x-11	*	324-x-12	***
			[32-4051]	[268-3902]		[82-3935]	
Q54CU4	COLA_DICDI	11103aa	433-x-17	430-x-17	*	**	424-x-22
			[1175-8554]	[1257-8691]			[301-9409]
Q8R0W0	EPIPL_MOUSE	6548aa	515-x-8	515-x-8	*	**	***
			[2000-6548]	[2067-6529]			
Q9Y6R7	FCGBP_HUMAN	5405aa	1367-x-3	1201-x-3	*	1201-x-5	394-x-13
			[1000-5102]	[1100-4811]		[21-5405]	[444-5382]
P05790	FIBH_BOMMO	5263aa	1049-x-5	168-x-30	8-x-19	**	***
			[1-5247]	[152-5221]	[3362-3495]		
Q9UKN1	MUC12_HUMAN	5478aa	1548-x-3	1557-x-2	25-x-8	28-x-151	***
			[74-4719]	[446-3569]	[2049-2280]	[215-5123]	
Q8WXI7	MUC16_HUMAN	22152aa	156-x-61	156-x-61	156-x-17	**	153-x-61
			[12038-21555]	[12047-21567]	[12420-15000]		[12046-21559]
Q6PZE0	MUC19_MOUSE	7524aa	652-x-9.6	163-x-36.4	*	**	***
			[1071-7372]	[1281 -7214]			
Q8WZ42	TITIN_HUMAN	34350aa	1082-x-4	28-x-6	10-x-26	**	395-x-28
			[22186-26525]	[11428-11596]	[11445-11686]		[20001-29694]

Analysis of the 12 proteins from the UniProtKB/Swiss-Prot database for which a tandem repeat of length ≥ 4000 aa has been detected by PTRStalker. For each protein (row) and each algorithm that returned at least a result (column) we report the longest TR found by each algorithm above the threshold of 100 aa. For each TR we report: the period -x- repeat number and the [interval spanned]. Fail to report is marked with "*". Note that HHRep and HHRepID are not listed here because they fail to report multi-repeating units, since they only report pairs of homologous substrings. Entries on the TRUST column marked "**" could not be completed because of excessive memory required (see Additional file 1). Entries in the RADAR column marked "***" correspond to absence of a TR cluster in the output, although many interspersed repeats may be found.

### Membrane proteins

Membrane proteins perform a wide range of biological functions including signal transduction and molecular transport. Here we report the behavior of PTRStalker in detecting fuzzy tandem repeats in two families of well known membrane proteins: the *Urea Transport Channels *and the *Chloride Channels*.

#### *Urea transport channels*

Transmembrane transport protein biology is key to the understanding of many critical biological process such as absorption and distribution of drugs within the human body. Transport proteins are basic component of transmembrane channels and often exhibit interesting symmetries at the structural level. Since experimental structural resolution of membrane proteins is difficult one would like to extract as much information as possible from the analysis of sequence data. In this set of experiments we tested the ability of PTRStalker in detecting blindly known repetitive structures in proteins of the Urea Transporter (UT) family [35]. Many members of the UT family are known to have a dimeric structure, but the level of amino acid identity between homologous subsequences is rather poor. Here we employ the metric based on the BLOSUM30 substitution matrices. For the purpose of detecting long fuzzy dimeric structures in proteins the tools XSTREAM and T-REKS proved unsuitable, therefore we tested PTRStalker against the tools HHRep, HHRepID, TRUST, and RADAR. Results in Table 5 show that all four methods could detect some dimeric structure of the four UT proteins under examination. In the case of UT-A1 [Mus musculus], PTRStalker attains a notably longer alignment w.r.t those reported by the three alternative methods, therefore giving a result more consistent with the known structure of that protein. On UT-A1 [Homo sapiens], TRUST and HHRepID gives a longer alignment. RADAR failed to highlight the dimeric structure.

**Table 5:** Analysis of proteins belonging to the Urea Transporter (UT) family

Protein			Tandem repeats found by				
**ID**	**Acc #**	**Length**	**PTRStalker**	**HHRep**	**HHRepID**	**TRUST**	**RADAR**

dvUT	ABM28909	337aa	[14-165]	[11-91]	[2-138 ]	[11-139]	5-x-44
			[177-323]	[174-254]	[141-286]	[140-303]	[23-269]
apUT	YP_001969475	300aa	[17-136]	[2-140]	[2-138]	[21-128]	[45-129]
			[153-284]	[156-288]	[156-286]	[175-278]	[199-277]
mUT-A1	AAM00357	930aa	[4-452]	[63-337]	[65-493]	[41-421]	5-x-52
			[467-918]	[532-800]	[533-916]	[422-866]	[117-775]
hsUT-A1	AAL08485	920aa	[4-320]	[50-338]	[87-490]	[102-564]	6-x-24
			[403-763]	[519-800]	[548-906]	[565-916]	[189-721]

dvUT = [D. vulgaris DP4], apUT =[A. pleuropneumoniae AP76], mUT-A1 = [Mus musculus], and hsUT-A1 = [Homo sapiens]. For each protein (row) and for each algorithm that returned at least a result (column) we report the longest fuzzy repeated subsequence detected by each algorithm by giving start and end position of homologous segments. Note that XSTREAM and T-REKS are not listed here because they are unsuitable for detecting long fuzzy dimeric structures.

#### *Chloride channel ClC-0*

CLC Channels are a family of membrane proteins whose major action is to translocate chloride ions across cell membranes. They have been subject to intense study since the cloning and identification of this protein in the species Torpedo marmorata (marbled electric ray) in 1990 [36]. This first identified protein, denoted CLC-0 (UniProtKB locus CICH TORMA, accession number P21564), is 805 aa long. It is divided into a pore domain in the region [49-507] and a cytoplasmatic domain in the region [508-805]. The pore domain has a diadic structure made of 18 alpha-helices organized in two symmetric groups of 9 alpha-helices each. The cytopalsmatic domain contains two CSB subdomains in positions [543-601] and [719-776]. Our hypothesis is that by analyzing just the amino acid sequence we can gain insight into the symmetries present in the structure of the protein (at least at a high level). We submitted the ClC-0 sequence to PTRStalker, XSTREAM, T-REKS, HHrep, HHrepID, TRUST, and RADAR. As shown in Table 6, HHrep identifies two homologous regions in positions [542-597] and [718-770] which coincide almost exactly with the CSB subdomains and two (short) partially overlapping domains that do not correspond to the global symmetry of the pore domain. Similarly TRUST reports shorter TR than those corresponding to known (global) symmetries of the protein. Instead, PTRStalker discovers a tandem structure in positions [8-289] [291-575] that covers most of the pore domain respecting its symmetry, and a tandem structure in positions [517-627] [628-800] that extends the two CSB subdomains. XSTREAM, T-REKS and HHrepID could not find any repetitive structure. RADAR and TRUST do not highlight any of the global known symmetries of ClC-0.

**Table 6:** Analysis of the CIC-0 protein belonging to the Chloride Channel family

Protein			Tandem repeats found by			
**ID**	**Acc #**	**Length**	**PTRStalker**	**HHRep**	**TRUST**	**RADAR**

CLC-0[CICH_TORMA]	P21564	805aa	[8-289]	[119-220]	[82-195]	[102-154]
			[291-575]	[174-260]	[196-303]	[161-205]
						[214-265]
			[517-627]	[542-597]	[447-483]	[329-353]
			[628-800]	[718-770]	[484-527]	[386-407]
					[528-564]	

For each algorithm that returned at least a result we report the fuzzy repeated subsequences detected by giving its start and its end position. Note that XSTREAM, T-REKS, and HHrepID are not listed here because they could not find any repetitive structure.

### Analysis of UniprotKB/Swiss-Prot

PTRStalker has been run to analyze the database in order to report Steiner Tandem Repeats with edit distance where the level of similarity between the repeated copies and the motif was greater than or equal to 0.7 i.e. error level *μ *= 0.3). The output of PTRStalker has been stored in a web-accessible database. The website takes as input the accession number of a sequence and shows a table containing all the TRs found in such a sequence. For every TR it displays the start and end position, the length of the motif, the number of repeats, the total length and the consensus string. The output can be filtered by range of values for several features of the TR (total length, motif length, number of repetitions).

**Figure 2:**
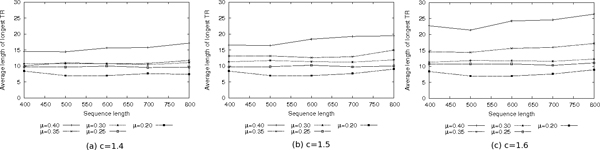
Average length of the longest TR found in shuffled sequences when using edit distance.

This analysis provides an indirect evaluation of PTRStalker with respect to other tools: in [17] T-REKS and other programs are compared by analyzing an old version of the Swiss-Prot database (Release of January 2009), which contained 356, 232 sequences. Among the four evaluated tools (T-REKS, Internal Repeats Finder [2], XSTREAM [16], and TRED [22]), T-REKS is the one that provided the best results by identifying 33, 780 sequences as containing TRs. The definition of TR used by T-REKS is the following: total length of TRs greater or equal to 14 residues (nine residues for homorepeats) and *P_sim _*≥ 0.7 (average level of similarity between copies and consensus). A direct comparison between PTRStalker and T-REKS would require the analysis of the same version of the Swiss-Prot database. Nevertheless, an indirect comparison can be performed by counting the percentage of sequences containing TRs (found with a given program) against the total number of analyzed sequences (we assume that such percentage is independent from the specific version of the used database). Let us call *P_TR _*such value. T-REKS, according to the numbers reported above and derived from [17], obtains *P_TR _*= 9.48%, while PTRStalker registers a value of *P_TR _*equal to 19.53% (for PTRStalker, we counted only the sequences for which a TR not shorter than 14 residues has been found, independently from the nature of the TR, i.e. homorepeats or not). Experiments shown in Figure 2 indicate that for the parameter setting used (c = 1.5, *μ*=0.3) the length 14 is above the average length of the longest TR in shuffled sequences.

### Statistical significance

In our algorithm several parameters have to be set, the most important ones are the error threshold *μ*, the slackness parameter *z *and the metric used (i.e. type of similarity matrix) that influences the value of *E*[*C*]. Since an improper configuration may result in detecting a distribution of fuzzy TRs (FTRs) indistinguishable from the distribution of FTRs one could derive from a set of random sequences, we need to perform a statistical significance test to validate the configuration of parameters. In particular, we concentrate on the distribution of the longest FTRs found in a population of biological strings (sampled from UniprotKB/SwissProt), w.r.t. a control set of random strings, with the same length and aa count composition.

The distribution of the longest FTR in a set of random sequences may in principle be modeled by an Extremal Value Distribution (usually Gumbel-type) via a parameter fitting phase that relies on extensive experimentation. This approach however relies on several assumptions and approximations requiring experimental verification on the actual data. In order to reduce the assumptions needed we prefer to use a non-parametric test that does not assume that the distribution under investigation has some specific property, thus resulting in more robust conclusions. We apply (with some modifications, and simplifications) a methodology described in [37,38] that, in turns is based on the Wilcoxon signed rank test of significance [39].

The Wilcoxon signed rank test allows to accept/reject the hypothesis that the distribution of differences among two series of paired scalar observations has a zero mean (null hypothesis). Being a non-parametric test, we can apply it without the need of strong hypothesis on the shape of the distribution under testing. The rational behind the test is that under the null hypothesis, the longest of the two paired longest FTR measurements is equally likely to be drawn from either sequence, thus the mean of the distribution of the differences is close to zero. In other words, if the longest FTR found by PTRStalker in biological sequences is not affected by a random shuffling of the input sequence, than we expect that the distribution of the difference of measurements (of the length of the longest FTR) has a zero mean. On the other hand, a mean value far from zero implies that the distributions from which the two sequences of scalar values are drawn are significantly different. A p-values for the Wilcoxon signed rank test below the threshold of *p *= 0.05 allows us to reject the null hypothesis and conclude that the longest FTR found in the biological sequences with a particular parameter setting are statistically significant. We report in Table 7 the *p*-values of the one-tailed variant of the test.

**Table 7:** P-values for the Wilcoxon signed rank significance test (one tailed test)

Length Class	P-value (1-tail)
300	1.5967199936708E-3
400	2.0342486747627283E-2
500	2.4673807804867205E-4
600	6.36671915517767E-7
700	1.8930737304578085E-5
800	1.8821140025488957E-5
900	7.568768556122764E-6
1000	5.4931640625E-4

We determine the significance of the fuzzy tandem repeats found using PTRstalker with the specific parameter setting (*μ *= 0.3, *c *= 1.5, *z *= 1, *BLOSUM*50).

The test is organized as follows. We subdivide the UniprotKB/SwissProt sequences in classes of length and we select the classes corresponding to lengths *l *= 400, 500, 600, 700, 800, 900, 1000. We test each length class separately. In each class, except class 900 and 1000, we randomly select 100 sequences that are shuffled to produce the control data set. In class 900 we select 81 sequences and in class 1000, 23 sequences, since these are all the sequences in those length class present in the database. The pairs of sequences (original and shuffled) are processed by PTRStalker and the length of the longest FTR found in both cases is returned as a matched pair to be fed to the Wilcoxon signed rank test. Since PTRStalker has a lower limit of 6 aa for the returned FTR, when one of the two runs fails to report a FTR we set it to the value 6 by default. When both runs fail to report a FTR we consider it a tie and is thus excluded in the testing, as required by the Wilcoxon procedure.

As we can see from the results reported in Table 7, the test gives p-values always below the 0.05 threshold for each length class considered.

### Tuning of parameters

The most important parameters that regulate the operation of PTRStalker are *μ*, *c*, and *z *(note that the latter makes sense only when the distance definition is BLOSUM-based). A set of experiments has been carried out to study the effects of such parameters in finding TRs. We built a set of sequences belonging to the following classes of length: 400, 500, 600, 700, 800. Each class contains 100 sequences and each sequence has been generated by shuffling a real sequence of UniprotKB/SwissProt with same length (this ensures that the resulting sequences have the same properties of biological sequences in terms of aa count).

Figure 2 shows the average length of the longest TR found in every sequence of the set, when using edit distance. As expected, for a given value of *c*, the average length of the longest TR depends on the value of *μ *when the value of *c *increases, the length of the TRs increases as well. Varying the value of *c *has a similar effect, this is particularly evident for the larger values of *μ*.

**Figure 3:**
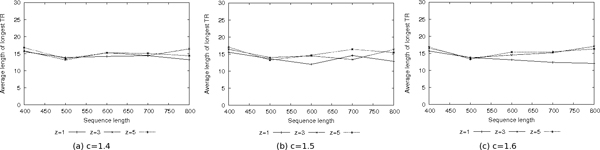
Average length of the longest TR found in shuffled sequences when using BLOSUM 50 based distance.

Similarly, Figure 3 shows the average length of the longest TR found in every sequence, when using a metric based on BLOSUM 50. More in detail, the results have been obtained keeping fixed the value of *μ *(0.3) to study the effects of *z *with three different values of *c*. The results show that, when using the BLOSUM-based metric, the length of the longest TR increases slightly with respect to the case when edit distance is used (curves for *μ *= 0.3 in Figure 2). The effects of both *c *and *z *are negligible when using the BLOSUM-based metric on the considered random sequences.

## Conclusions and future work

Discovering fuzzy tandem repeating units in amino acid sequences gives precious hints as to the internal protein organization and symmetries. However due to high level of protein sequence divergence this task is considered challenging even for relatively short sequences. In this paper we presented a new algorithm, PTRStalker, opportunely tuned for detecting amino acid tandem repeats within protein sequences. We proved that PTRStalker pushes forward the state of the art. Indeed, feeding PTRStalker with sequences from the UniProtKB/Swiss-Prot database did allow us to detect novel repetitive structures not captured by other state-of-the-art tools. In particular, we could find a notable long fuzzy TR in Human Titin that several competing methods missed. For Chlorine channel protein ClC-0, we showed that PTRStalker can detect general symmetries not detected by competing methods.

We believe that a tool such as PTRStalker can be used to extract valuable structural hints from protein sequences for which no tertiary structure (determined via X-ray or NMR) is available.

Future work will aim at comparing the relative power of different amino acid metric spaces within the PTRStalker framework. In particular we are interested in those based on PAM matrices [8] and those based on the vector space mapping approach [9]. Moreover, we will study the correlations between structural classifications of protein families (e.g. those in SCOP and CATH) and the fuzzy tandem repeats found in primary sequences.

## Competing interests

The authors declare that they have no competing interests.

## Authors' contributions

AV conceived of the application tool and the web data base, participated in its design and development, and helped to draft the manuscript. MER participated in the design of the application, performed the testing and debugging phases, performed experiments, and helped to draft the manuscript. MP exercised general supervision, conceived and performed the experiments, drafted the final manuscript, and exercised general supervision. All authors read and approved the final manuscript.

## Supplementary Material

Additional file 1 **Ab initio detection of fuzzy amino acid tandem repeats in protein sequences - supplementary information -.** Description of the parameters and settings used in testing the competing sw for TR detection. Click here for file
