# Supplementary material for: Ab initio detection of fuzzy amino acid tandem repeats in protein sequences
Source: BMC Bioinformatics. 2012 Mar 21;13(Suppl 3):S8. doi: 10.1186/1471-2105-13-S3-S8 (PMC3402919; doi:10.1186/1471-2105-13-S3-S8)
Supplement: Additional file 1 — Ab initio detection of fuzzy amino acid tandem repeats in protein sequences - supplementary information -. Description of the parameters and settings used in testing the competing sw for TR detection. [file 1471-2105-13-S3-S8-S1.pdf]

Ab initio detection of fuzzy amino acid tandem repeats in protein sequences
# – supplementary information –

Marco Pellegrini*[1] and M. Elena Renda[1] and Alessio Vecchio[2]

[1]Istituto di Informatica e Telematica, CNR – Consiglio Nazionale delle Ricerche, Pisa I-56124 , Italy
[2]Dipartimento di Ingegneria dell'Informazione, Università di Pisa, Pisa I-56122 , Italy

Email: Marco Pellegrini*- marco.pellegrini@iit.cnr.it; M. Elena Renda - elena.renda@iit.cnr.it; Alessio Vecchio - a.vecchio@ing.unipi.it;

*Corresponding author

Parameter setting: TRUST

The TRUST utility is available in two forms. As web interface at:

`http://www.ibi.vu.nl/programs/trustwww/`

and as java code available for download from the same website. Results for Tables 5 and 6 have been obtained from the website tool with the following parameters: Gap open penalty = 8, gap extension penalty = 2, substitution matrix BLOSUM62, Force including local alignments = no, low-complexity filtering = yes, which are the standard parameters.

In order to process longer sequences of Table 4 we have used the downloaded code locally on a MacBook Pro, with Processor Intel Core 2 Duo, 2.4 GHz, Cache 3 MB, Memory 4 GB. The code was executed with the options:

```
java -Xms2G -Xmx3G -cp . nl.vu.cs.align.SelfSimilarity -fasta <InputFilename.fasta>
-matrix BLOSUM50 -gapo 8 -gapx 2 -noseg
```

For entries of Table 4 marked (**) the execution count not be completed for an out-of-memory fault even when allowed memory was raised to the available physical memory.

Parameter setting: XSTREAM

XSTREAM is available as on-line web interface at

http://jimcooperlab.mcdb.ucsb.edu/xstream/

It has been run with standard (default) parameters.

Parameter setting: T-REKS

T-REKS is available as on-line web interface at

http://bioinfo.montp.cnrs.fr/?r=t-reks

It has been run with standard (default) parameters. In particular the similarity parameters (among pairs of consecutive repeats) is set to 70% which is the lowers value allowed.

Parameter setting: HHRep

HHRep is available as on-line web interface at

http://toolkit.tuebingen.mpg.de/hhrep

It has been run with standard (default) parameters.

Parameter setting: HHRepID

HHRepID is available as on-line web interface at

http://toolkit.tuebingen.mpg.de/hhrepid

It has been run with standard (default) parameters.

Parameter setting: RADAR

RADAR is available as on-line web interface at

http://www.ebi.ac.uk/Tools/Radar/

It has been run with standard (default) parameters. The online web interface has a limit of 10000 aa to the length of the input sequences. Sequences in table 5 of length greater then 10000 have been split into smaller substrings (of 10000 aa) and analyzed separately.
